# Parallelized Seeded Region Growing Using CUDA

**DOI:** 10.1155/2014/856453

**Published:** 2014-09-22

**Authors:** Seongjin Park, Jeongjin Lee, Hyunna Lee, Juneseuk Shin, Jinwook Seo, Kyoung Ho Lee, Yeong-Gil Shin, Bohyoung Kim

**Affiliations:** ^1^SW Content Research Laboratory, Electronics and Telecommunications Research Institute, 218 Gajeong-Ro, Yuseong-Gu, Daejeon 305-700, Republic of Korea; ^2^School of Computer Science & Engineering, Soongsil University, 369 Sangdo-Ro, Dongjak-Gu, Seoul 156-743, Republic of Korea; ^3^Department of Brain and Cognitive Sciences, Seoul National University, 1 Gwanak-Ro, Gwanak-Gu, Seoul 151-742, Republic of Korea; ^4^Department of Systems Management Engineering, Sungkyunkwan University, 2066 Seobu-ro, Jangan-gu, Suwon-si, Gyeonggi-do 440-746, Republic of Korea; ^5^School of Computer Science and Engineering, Seoul National University, 599 Kwanak-ro, Kwanak-gu, Seoul 151-742, Republic of Korea; ^6^Department of Radiology, Seoul National University Bundang Hospital, Seoul National University College of Medicine, 82 Gumi-ro 173 Beon-gil, Bundang-gu, Seongnam-si, Gyeonggi-do 463-707, Republic of Korea; ^7^Department of Radiology, Seoul National University Bundang Hospital, 82 Gumi-ro 173 Beon-gil, Bundang-gu, Seongnam-si, Gyeonggi-do 463-707, Republic of Korea

## Abstract

This paper presents a novel method for parallelizing the seeded region growing (SRG) algorithm using Compute Unified Device Architecture (CUDA) technology, with intention to overcome the theoretical weakness of SRG algorithm of its computation time being directly proportional to the size of a segmented region. The segmentation performance of the proposed CUDA-based SRG is compared with SRG implementations on single-core CPUs, quad-core CPUs, and shader language programming, using synthetic datasets and 20 body CT scans. Based on the experimental results, the CUDA-based SRG outperforms the other three implementations, advocating that it can substantially assist the segmentation during massive CT screening tests.

## 1. Introduction

Image segmentation, which identifies features or objects in a 2D image or in 3D volume data, is a challenging data-dependent task in medical image analysis. Although fully automatic segmentation, which identifies objects automatically without user interaction or feedback, could serve as an optimal solution for computer aided diagnosis (CADx), it does not often guarantee accurate segmentation. In contrast, semiautomatic segmentation involving user interaction provides faster and more accurate segmentation. There have been substantial researches on semiautomatic segmentation methods [1–14]. Among these studies, seeded region growing (SRG) is one of widely used segmentation methods for identifying relatively homogeneous objects such as lungs and colons due to its computational simplicity [2, 3, 5, 8].

SRG detects all the connected voxels, which satisfy the predefined condition (e.g., intensity threshold). Given a set of seed points specified by users, their neighboring voxels are examined to determine if they have characteristics (e.g., intensity value) that are similar to those of the seed points. The neighboring voxels with sufficient similarity are collected and then added to the segmented region. This process continues by examining the neighbors of the added voxels for further collection. The algorithm stops when no more voxels are left for examination.

The computation time of SRG is theoretically proportional to the number of voxels in the 3D volume data (or the number of pixels in a 2D image) corresponding to the final segmented region because the segmented region is expanded by one voxel at a time. SRG identifies a small region at an interactive speed; however, it requires up to over ten seconds to segment a relatively large region consisting of more than 30 mega voxels, even on a high-end PC with, for example, a 2.83 GHz quad-core processor and 8 GB of memory.

In order to accelerate the SRG computation and to make it independent of the segmented region size, a parallel processing technique can be applied. Modern CPUs have up to 16 cores (commonly dual-core or quad-core), and highly parallel computing devices such as graphics processing units (GPUs) have more than hundreds of cores [15]. Open multiprocessing (OpenMP) can be used as a method for parallel processing on CPU. OpenMP is an application programming interface that supports multiplatform shared-memory parallel programming in C, C++, and Fortran [16]. With the advent of commodity inexpensive multicore processors and corresponding OpenMP-capable compliers, OpenMP has gained the popularity [17–19].

GPUs have developed very quickly in recent years, now being much more powerful than modern CPUs [15]. GPUs are essentially massive parallel-processing devices with many small processing units. Therefore, they can be successfully applied to highly parallel computing problems. GPUs became programmable with the introduction of shader languages (e.g., High Level Shader Language, or HLSL; OpenGL Shading Language, or GLSL; and C for Graphics, or Cg). However, shader language programming is complicated as it requires prior knowledge of computer graphics. With the advent of new technologies such as Compute Unified Device Architecture (CUDA) [20], it has become more viable to perform sophisticated algorithms on GPUs with relatively simple programming methodologies.

There have been reported researches on parallelizing segmentation algorithms using GPUs. Yang and Welch [21] used register combiners to perform thresholding and basic convolutions on two-dimensional color images. Their GPU implementation with NVIDIA Geforce4 demonstrated a 30% speedup over a CPU implementation on 2.2 GHz Intel Pentium 4 CPU. Viola et al. [22] proposed a three-dimensional segmentation method using thresholding combined with an interactive visualization, observing nearly an eight-fold speedup over a CPU implementation.

Rumpf and Strzodka [23] introduced the implementation of level set segmentation on GPU. They performed 2D image segmentation using a 2D level-set equation with intensity and gradient forces. Lefohn and Whitaker [24] extended that work to implement the first 3D level-set segmentation on GPU. Their implementation allowed users to control the curvature of the evolving segmentation interactively and supported a more complex evolution function for more accurate segmentation. These implementations were not faster than highly optimized CPU implementations due to the computation of level-set values for the entire image. Lefohn et al. [25, 26] enhanced their earlier work by using an optimized 3D level-set method. Their methods computed level-set values only on the boundary region of the level-set contour instead of the entire image, resulting in a 10–15x speedup over highly optimized CPU implementations. Sherbondy et al. [27] also presented a GPU-based 3D segmentation method which used the depth culling technique for conditional execution in computing level-set values on the boundary region of the level-set contour.

Only a few approaches have been proposed that utilized the CUDA technology to implement segmentation algorithms. Vineet and Narayanan [28] presented a fast implementation of the push-relabel algorithm for mincut/maxflow algorithm for graph-cuts using CUDA. They used 640 × 480 size benchmark images and 1024 × 1024 size synthetic images on an NVIDIA GTX 280 for the performance comparison. Their implementation was 10–12 times faster than the best sequential algorithm reported. Pan et al. [29] implemented SRG in CUDA. They compared the SRG implementations in CUDA, Cg, and serial CPU. In their experimental results, the CUDA implementation showed a slight improvement in efficiency compared to the Cg implementation with large data, but it was 1.6 times faster than the serial CPU implementation.

In this paper, we propose a novel method for parallelizing the SRG algorithm using the CUDA technology. The segmentation performance of the proposed method is assessed in comparison with SRG implementations on single- and quad-core CPUs and shader language programming, using synthetic datasets and medical CT datasets.

## 2. Methods

### 2.1. CUDA Architecture

The GPUs are especially suited to address parallel processing problems wherein the same program is executed on many data elements in parallel. Such data-parallel processing maps data elements (e.g., pixels in 2D image processing or voxels in 3D rendering) to parallel processing threads. Recently, CUDA was introduced by NVIDIA as a general purpose computing architecture which utilizes the parallel processing units in GPUs to solve many complex computational problems [15]. By supporting various high-level programming languages, CUDA enables developers familiar with standard programming languages such as C to adapt to the parallel programming using CUDA at a low learning cost.

**Figure 1:**
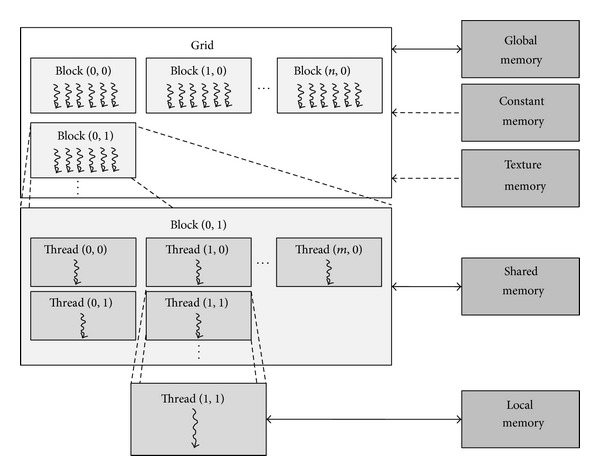
Thread and memory hierarchy of CUDA architecture.

The CUDA parallel programming is based on Single Instruction Multiple Thread (SIMT), in which multiple threads execute the same single instruction. When a function is called, it is executed *N* times in parallel by *N* different CUDA threads. The CUDA threads are organized in a hierarchical way as follows (Figure 1).

- Thread: each thread executes a given function and has a unique index, called thread id.
- Thread block: threads are grouped to a thread block. Thread blocks are required to be executed independently—they should be possible to be executed in any order, in parallel, or in succession.
- Grid: a group of thread blocks are organized into a grid. A single grid is assigned to a single GPU; thus a grid can be executed on one GPU, not on multiple GPUs.

Those hierarchical CUDA threads, during their execution, access data from multiple (read-write) memory spaces which are also hierarchically organized matching to the thread hierarchy. The memory hierarchy is as follows (Figure 1).

- Local memory: each thread has its private local memory.
- Shared memory: each thread block has shared memory accessible by all the threads in the block. The shared memory has the same lifetime as its corresponding block. Threads in a block can cooperate with one another by sharing the data through the shared memory.
- Global memory: all threads have access to the same global memory. The global memory acts as the buffer for interblock communication for all the threads in the same GPU.

There are two additional read-only memory spaces accessible by all threads and constant and texture memory spaces (Figure 1). The global, constant, and texture memory spaces are optimized for their specific memory usages. Based on these thread and memory hierarchies, the CUDA architecture brings out both high throughput and flexibility for the SIMT paradigm.

### 2.2. SRG Parallelization Using CUDA Technology

The SRG parallelization using the CUDA technology involves three steps: volume/mask data loading, 3D thresholding, and 3D region growing.

#### 2.2.1. Volume/Mask Data Loading

**Figure 2:**
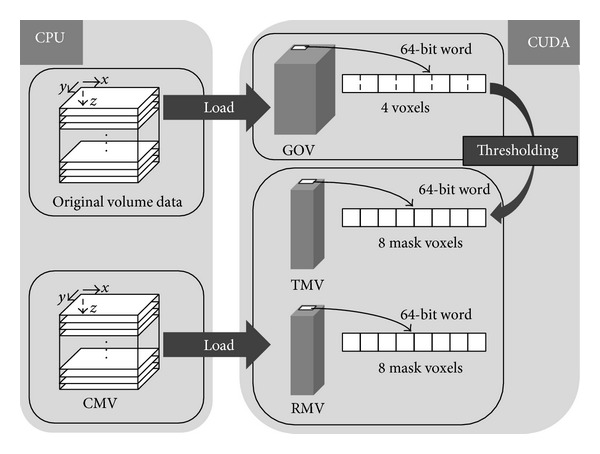
Loading of the original volume data and mask volume data from CPU main memory to CUDA global memory. GOV is GPU original volume, CMV is CPU mask volume, TMV is threshold mask volume, and RMV is region mask volume.

Original volume data and mask volume data for storing the segmentation result are loaded from the CPU memory to the CUDA global memory. The original volume data (CT or MR data in medical field) typically consists of a series of 2D slice images. Each 2D slice image has a bit depth of 12 bits/pixel, and each pixel is packed on a two-byte boundary with four padding bits. The CUDA device is capable of reading a 32-, 64-, or 128-bit word from the global memory in a single instruction [15]. From a preliminary test, a 64-bit word showed the best computation performance. Therefore, the original volume data and the mask volume data are both loaded into the global memory of a 64-bit word. As the original volume data consists of 16-bit voxels, four successive voxels along the *x*-axis are stored in a single word in the CUDA global memory (GPU original volume, or GOV) (Figure 2).

The mask volume data in the CPU memory (CPU mask volume, or CMV) has the same resolution as the original volume data and consists of 8-bit binary voxels, 0 for “nonsegmented” or 255 for “segmented.” CMV is loaded into two sets of 3D memories in the CUDA global memory, denoted by TMV (threshold mask volume for storing tentative thresholding result) and RMV (region mask volume for storing final segmentation result). Each voxel of TMV and RMV is of a 64-bit word, where eight successive mask voxels along the *x*-axis in CMV are stored in a single word of TMV and RMV (Figure 2). In this way, four voxels in the original volume and eight mask voxels in the mask volume can be simultaneously processed in a single CUDA operation.

#### 2.2.2. 3D Thresholding

After loading the data into the CUDA global memory, a 3D thresholding is tentatively preformed using a user-specified seed point. The result is then stored into TMV. In the CUDA technology, a thread is a processing unit in charge of operations for a voxel in the CUDA global memory. For every voxel in the entire volume, its corresponding thread reads its intensity value. If the current voxel has an intensity value similar to that of the seed point under a user-specified similarity criterion, its mask value is set to 255 in TMV, indicating that the voxel is processed in the next 3D region growing step; otherwise, the mask value is set to 0, indicating that the voxel is not be processed further.

#### 2.2.3. 3D Region Growing

After the 3D thresholding is done, a 3D region growing is performed by referencing only the two mask volumes, TMV and RMV. As TMV already contains mask results thresholded by a similarity criterion, the original volume data does not have to be referenced in this 3D region growing operation.

RMV for storing the final segmentation result is initialized so that only the mask value for the seed point is set to 255. For each voxel, its corresponding thread reads a mask value from RMV. If the current RMV mask value is 255 (already segmented), the thread reads a mask value from TMV (Pseudocode 1). If the TMV mask value is also 255, each RMV mask value of 6 neighboring voxels is updated to be bitwise-OR'ed with the corresponding TMV mask value. After the update of RMV mask values of the 6 neighboring voxels, the current TMV mask value is set to 0, preventing the current voxel from being processed in later iterations. In this way, the segmented region starting from the seed point grows along the *x*-, *y*-, and *z*-axes. In Pseudocode 1, *threshold*_*mask*_*value* (*x*, *y*, *z*) and *region*_*mask*_*value* (*x*, *y*, *z*) are functions referencing the mask values at the voxel position (*x*, *y*, *z*) from TMV and RMV, respectively. These are defined as follows:

(1) threshold_mask_value(x,y,z)  =(x%8)th  8-bit  value  of  TMV(⌊x8⌋,y,z),

(2) region_mask_value(x,y,z)  =(x%8)th  8-bit  value  of  RMV(⌊x8⌋,y,z).

**Pseudocode 1:**
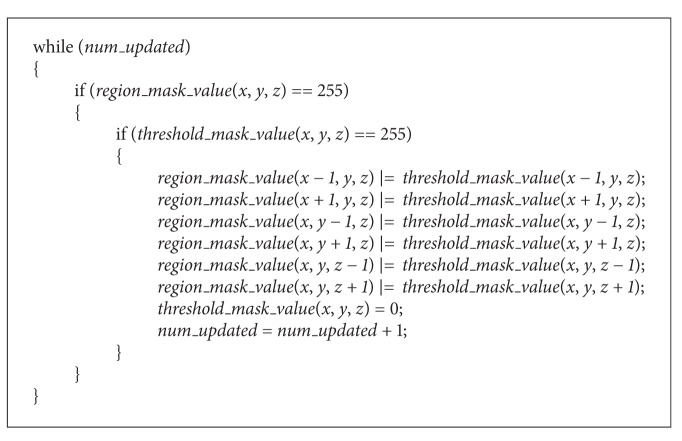
Pseudocode of 3D region growing. num_updated is the number of updated voxels in the current iteration.

The aforementioned mask volume update is iterated until there is no change in the RMV mask values. The counter variable,* num_updated,* counts the number of threads which update the RMV mask values.* num_updated* is initialized to 0 in each iteration. When a thread updates the RMV mask values of 6 neighboring voxels,* num_updated* increases by 1. If* num_updated* is 0 after an iteration, indicating no change in RMV mask values, the 3D region growing is terminated.

### 2.3. Advantage of CUDA Programming over Shader Language Programming

The SRG algorithm can be implemented using shader language programming in a similar way as in using the CUDA technology. After the volume/mask data loading and the seed point specification, the 3D region growing is performed with iterative mask volume updates. Because the GPU texture memory in shader language programming cannot be used both for “read” and “write” operations, it is required to allocate two texture memories, one for “read” and the other for “write.” The two texture memories switch their roles as “read” and “write” memories after each iteration of mask volume update.

In shader language programming, a pixel shader processor is a processing unit in charge of operations for a texel in the GPU texture memory. For every voxel, its corresponding pixel shader processor reads and writes mask values. If the mask value of the current voxel is 255 (already segmented), the corresponding pixel shader processor copies 255 to the corresponding position in the writing mask memory. When the current voxel has a mask value of 0 and its intensity is similar to that of the seed point under a user-specified similarity criterion, a bitwise-OR value of the mask values of its six neighboring voxels is set to the position of the current voxel in the writing mask memory. In this way, a voxel having the intensity similar to that of the seed point and having a segmented voxel in its 6-neighborhood is incrementally added to the segmented region.

The SRG parallelization using shader language programming has a couple of limitations due to the fundamental constraints of shader language programming. It would be ideal to determine if a voxel is included in the segmented region in a single reading and a single writing operation. However, as a pixel shader processor can update only its corresponding single voxel value, a pixel shader processor for each nonsegmented voxel has to read 6 neighboring mask voxels to update only its corresponding* single* mask value in each iteration. In addition, because the pixel shader processor, following the graphics pipeline, should write its corresponding voxel value to the GPU texture memory in every iteration; it should write a mask value to the writing mask memory even for previously segmented voxels, resulting in redundant writing operations.

Those limitations of shader-language-based SRG parallelization can be overcome by using the CUDA technology. In contrast to the pixel shader processor with the constraint of single voxel writing in shader language programming, a CUDA thread can write multiple mask values, so that the thread can update as many as six mask values for 6 neighboring voxels at once. This multiple writing can faithfully simulate the operation of the original SRG algorithm. In addition, the CUDA-based SRG parallelization can avoid processing the previously segmented voxels further in an iteration during the 3D region growing operation.

## 3. Experimental Results

We tested the proposed CUDA-based SRG parallelization on an Intel Core i5-3570 desktop system with a 3.4 GHz quad-core processor and 8 GB of memory. The system was also equipped with a GeForce GTX 285 GPU with 1 GB of memory. For the performance comparison, we also implemented the SRG using HLSL, which is one of commonly used shader languages, on GeForce GTX 285. CUDA 2.3 and HLSL Shader Model 4.0 with DirectX 10.0 were used. In addition, we implemented the CPU-based SRG on single-core and quad-core architectures. We used OpenMP library 4.0 for the CPU-based SRG on the quad-core architecture. We tested the four methods (CUDA, HLSL, single-core CPU, and quad-core CPU methods) with both synthetic and patient data. The computation times of the four methods with the patient data were compared using Friedman tests with posthoc tests with a *P* value threshold of 0.05.

### 3.1. Synthetic Data Results

The synthetic data included three datasets of a cube, a cylinder, and a sphere. The volume sizes of the synthetic data were 512 × 512 × 512 in all cases. In order to demonstrate the effect of the size of segmented region on the computation performance, we varied the side length of the cube, the height and radius of the cylinder, and the radius of the sphere (Table 1).

**Figure 3:**
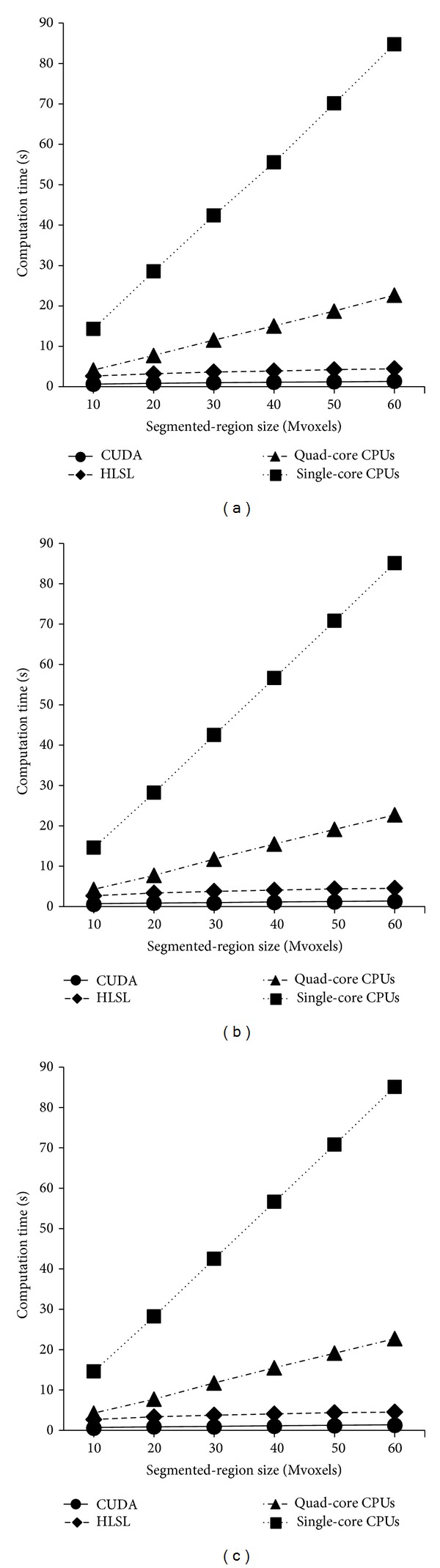
Computation time results for the (a) cube, (b) cylinder, and (c) sphere datasets.

Figure 3 shows the results of computation time in seconds, averaged over multiple tests. When the segmented-region size was 10 mega voxels (Mvoxels), the computation time increased in the order of the CUDA, HLSL, quad-core CPU, and single-core CPU methods. With an increment of 10 Mvoxels in the segmented-region size, the single-core CPU, quad-core CPU, HLSL, and CUDA methods exhibited an increment of 14.1 ± 0.5 (mean ± SD), 3.7 ± 0.3, 0.4 ± 0.1, and 0.1 ± 0.0 seconds, respectively, in their computation time.

As the segmented-region size increased, the computation times of the two CPU methods increased greatly, whereas those of the HLSL and CUDA methods increased very slowly. Particularly, the CUDA method required nearly constant computation time regardless of the segmented-region size. These results were consistent for all the three synthetic datasets. The great increment in computation time for the two CPU methods was expected considering their fundamental principle of the voxel-by-voxel collection of neighboring voxels with sufficient similarity. In contrast, the minute or nearly no increment in computation time for the HLSL and CUDA methods is attributed to the fact that they have all the voxels in the volume be read at least once during each iteration. Thus, they are not affected by the number of segmented voxels (i.e., the segmented-region size), but they might be rather affected by the number of whole voxels.

**Figure 4:**
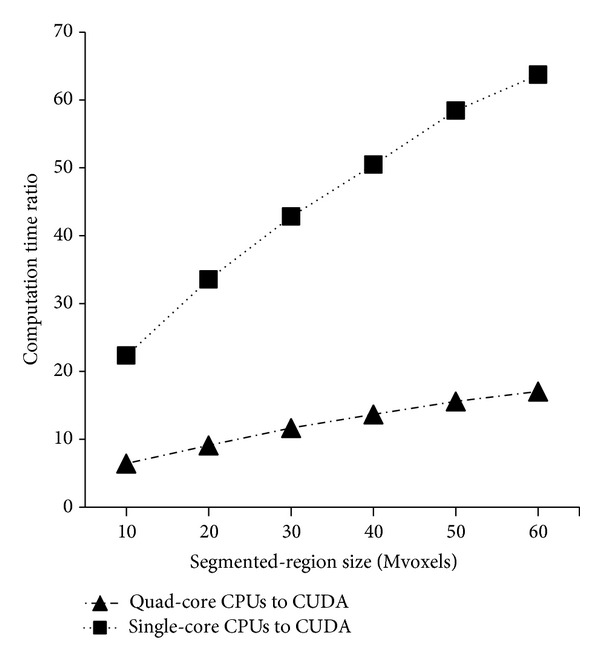
Computation time ratios of single-core and quad-core CPU implementations to that of CUDA implementation for the cube dataset.

The CUDA method required the least computation time for all the tested segmented-region sizes in all the three synthetic datasets. To visually represent the advantage of the CUDA method (the best GPU method in this study) over the CPU methods, we calculated the ratio of the computation time of the CPU method to that of the CUDA method. As all the three synthetic datasets exhibited a similar graph pattern, we only plotted the computation time ratio for the cube dataset (Figure 4). As the segmented-region size increased, the relative advantage of the CUDA method over the CPU methods increased, demonstrating a much greater increase in the computation time ratio for the single-core CPU than for the quad-core CPU.

### 3.2. Patient Data Results

**Figure 5:**
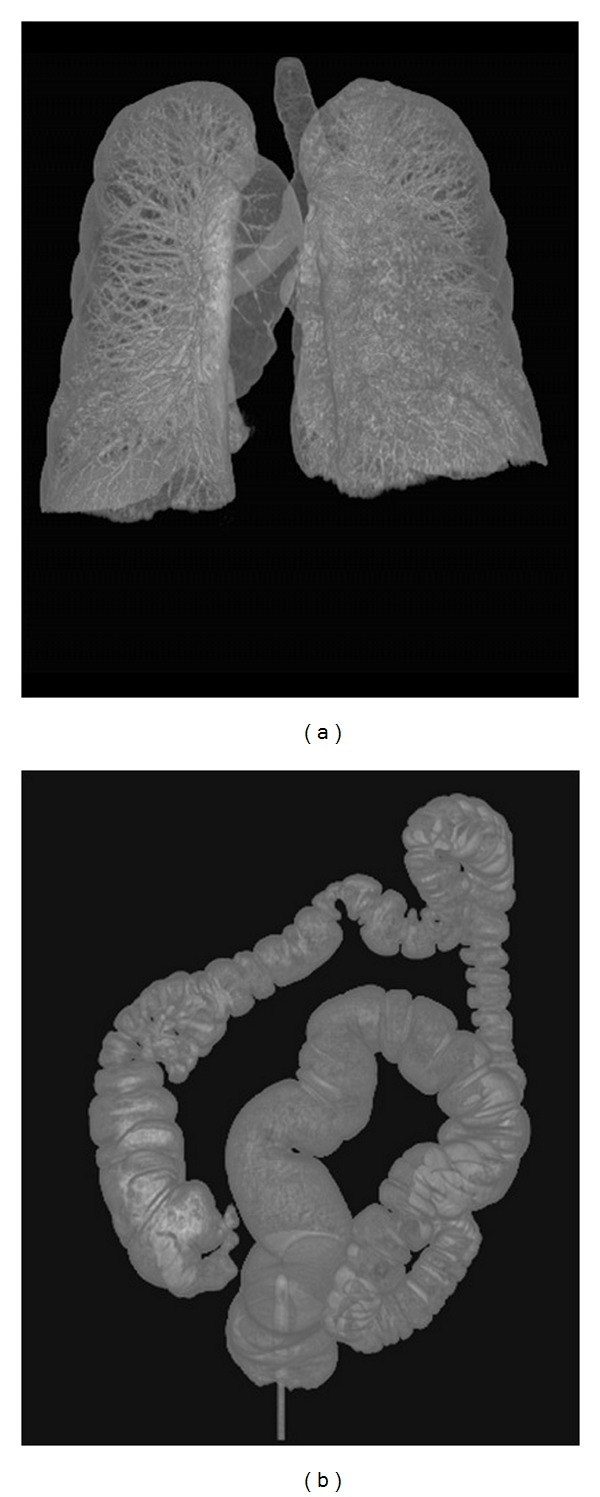
Volume-rendered images of a segmented (a) lung and (b) colon.

The patient data included 10 lung and 10 colon CT scans (Table 2).  Table 3 shows the results of the computation time in seconds and the number of iterations for the patient data. As the original SRG algorithm was not an iterative algorithm, the number of iterations was not included in the CPU implementations in Table 3.  Figure 5 shows volume-rendered images of a segmented lung and colon from our experiments.

For the lung CT scans, the computation time increased significantly in the order of the CUDA, HLSL, quad-core CPU, and single-core CPU methods (*P* < 0.01 for all pair-wise comparisons). For the colon CT scans, the computation time increased significantly in the order of the CUDA, quad-core CPU, single-core CPU, and HLSL methods (*P* < 0.01 for all pair-wise comparisons).

The HLSL method interestingly showed the worst performance for the colon scans even with its parallelized computation. The colon scans, compared to the lung scans, had a* smaller* segmented-region size (colon versus lung, 7.0 ± 3.1 (mean ± SD) versus 13.6 ± 1.8) but a* greater *number of iterations (337.1 ± 98.0 versus 145.1 ± 52.6 for the HLSL method). The single-core and quad-core CPU methods benefited directly from the smaller segmented-region size of the colon scans, exhibiting the less computation time for the colon scans. In contrast, the HLSL method, which is not so much affected by the segmented-region size as demonstrated in the synthetic data results, showed the increased iterations for the colon scans likely due to the structural complexity of the folding and crumpling colon. The CUDA and HLSL methods both required much more iterations for the colon scans than for the lung scans, resulting in the much more computation time for the colon scans. However, the CUDA method, which features the writing of multiple mask values and the avoidance of redundant writings, still exhibited the best performance for the colon scans as well as for the lung scans.

## 4. Discussion

When the segmented-region size was small in the synthetic data, the computation time increased in the order of the CUDA, HLSL, quad-core CPU, and single-core CPU methods. As the segmented-region size increased, the single- and quad-core CPU methods required dramatically increasing computation time, whereas the CUDA and HLSL methods exhibited a very slow increase in computation time. In particular, the CUDA method exhibited a nearly constant computation time regardless of the segmented-region size for the CUDA method. The performance of the original SRG algorithm is fundamentally affected by the segmented-region size, which may have been one factor. The CUDA method showed the best performance for all the tested segmented-region sizes in all the synthetic datasets. It also performed the best for the patient datasets of lung and colon CT scans.

Segmentation in lung and colon CT datasets has recently emerged as an important topic, as both examinations have rapidly gained support as screening tests in populations. From a practical viewpoint, it is very challenging to interpret such examinations of huge volumes in a timely manner. Therefore, any new technology would be welcomed if it is helpful in automating any part of the interpreting processes. The proposed CUDA-based SRG method, which demonstrated the best performance in our experiments, would be helpful in improving the performance of such an automated system. Another practical advantage of the CUDA method over a CPU method would be that its performance can be improved even more if it is used in conjunction with the designation of an ROI (region of interest). As shown in our study results, the performances of the two CPU methods highly depend on the segmented-region size regardless of the presence of such an ROI. Thus, the CPU methods would not benefit from the ROI designation which does not change the segmented-region size. In contrast, the CUDA method, the performance of which rather depends on the resolution of data (i.e., the number of whole voxels), may realize a substantial practical gain in the computational efficiency via the ROI designation.

Recent work was done by Pan et al. [29], in which seeded region growing was implemented using the CUDA technology. Comparing with Pan et al. [29], our method designed data structures to maximize the utilization of CUDA architecture. Their method read an 8-bit word from the global memory in a single instruction, whereas our method read a 64-bit word from the global memory in a single instruction. Due to the limitation of the number of the available threads, the data size handled by one thread determines the acceleration performance. Their method processed only one voxel per one thread, whereas our method processed eight voxels per one thread. Since the SRG algorithm refers the information of neighboring voxels, we developed the data structures to refer the current and neighboring voxels efficiently, which were concurrently stored in a 64-bit word data.

A direct comparison of the processing time between their work [29] and ours is likely unfair, as different graphics cards were used (i.e., Geforce 8500 GT in their work versus Geforce GTX 285 in ours). They segmented the heart, artery, and bone simultaneously using multiple seed points in an abdomen CT scan with a resolution of 512 × 512 × 289. For comparison, we segmented the lung in a lung CT scan with a resolution of 512 × 512 × 332, which had the closest number of slice images as their work. Considering that the number of slice images is greater in our experiment than in that of Pan et al. and the lung has a larger volume than the total volume of the heart, artery, and bone, the comparison is not favorable to ours, rather favoring that of Pan et al.. Our mean computation time was 0.53 s, while theirs was 12.88 s (2.26 s considering the 5.71x GPU performance increase from Geforce 8500 GT to Geforce GTX 285).

The proposed CUDA-based SRG method has a limitation. The method requires twice the amount of memory for the original volume data. It uses two sets of mask volume data to store the segmentation result. Each mask dataset with 8 bits per voxel amounts to half of the original volume data, and thus the two datasets requires exactly the same amount of memory storage for the original volume data. This additional storage is not likely negligible considering the limited amount of GPU memory space. However, the use of additional storage may be unavoidable considering the computational efficiency gain by the proposed CUDA method.

## 5. Conclusion

This paper proposed a method of parallelizing the SRG algorithm using the CUDA technology. The segmentation performance of the proposed CUDA-based SRG method was evaluated in comparison with the SRG implementations on single- and quad-core CPUs and shader language programming. By exploiting the feature of the CUDA technology of simultaneous writing to multiple mask voxels and by avoiding redundant writings, the proposed CUDA-based SRG outperformed the other three methods both for the synthetic and patient datasets. Considering its incomparable computational efficiency and its advantageous feature of a nearly constant computation time regardless of the segmented-region size, the proposed CUDA-based SRG can be advocated to substantially assist the segmentation during massive CT screening tests.

## Figures and Tables

**Table 1:** Synthetic datasets.

Segmented-region size (Mvoxels)	Cube	Cylinder		Sphere
	Side length (pixels)	Height (pixels)	Radius (pixels)	Radius (pixels)
10	219	238	119	136
20	276	298	149	171
30	316	342	172	196
40	347	376	188	215
50	374	406	203	232
60	398	432	216	247

**Table 2:** Patient datasets.

Patient data	Number of slice images	Segmented-region size (Mvoxels)
Lung	358.8 ± 14.6 (332, 376)	13.6 ± 1.8 (11.2, 16.5)
Colon	507.2 ± 31.5 (468, 580)	7.0 ± 3.1 (2.9, 13.2)

Note: data are means ± SD (minimum and maximum range) for 10 CT scans. The resolution of each CT image is 512 × 512.

**(a):** 

Patient data	Computation time (s)^a^			
	CUDA	HLSL	Quad-core	Single-core
Lung	0.6 ± 0.1 (222.8 ± 22.2)	4.5 ± 1.3 (145.1 ± 52.6)	8.7 ± 2.8 (n/a)	19.3 ± 2.4 (n/a)
Colon	1.3 ± 0.4 (461.1 ± 189.3)	13.2 ± 4.0 (337.1 ± 98.0)	4.2 ± 1.8 (n/a)	7.4 ± 3.9 (n/a)

**(b):** 

Patient data	*P* value						
	Overall	CUDA versus HLSL	CUDA versus quad-core CPUs	CUDA versus single-core CPUs	HLSL versus quad-core CPUs	HLSL versus single-core CPUs	Quad-core CPUs versus single-core CPUs
Lung	<0.01	<0.01	<0.01	<0.01	<0.01	<0.01	<0.01
Colon	<0.01	<0.01	<0.01	<0.01	<0.01	<0.01	<0.01

Note: ^a^data are means ± SD of the computation time (mean ± SD of the number of iterations) for 10 CT scans.
